# Adapting Novel Augmented Reality Devices for Patient Simulations in Medical Education

**DOI:** 10.7759/cureus.66209

**Published:** 2024-08-05

**Authors:** Seth M Alexander, Vince Friedman, Pirapat M Rerkpattanapipat, William A Hiatt, Jeremiah S Heneghan, Robert Hubal, Yueh Z Lee

**Affiliations:** 1 Health Sciences, University of North Carolina School of Medicine, Chapel Hill, USA; 2 Internal Medicine and Pediatrics, Vanderbilt University Medical Center, Nashville, USA; 3 Radiology, University of North Carolina School of Medicine, Chapel Hill, USA; 4 Simulations, BioMojo LLC, Cary, USA; 5 Medical Education, Renaissance Computing Institute, Chapel Hill, USA

**Keywords:** patient history, automated patient history collection, augmented reality (ar), natural language processing (nlp), simulation in medical education

## Abstract

Extended reality (XR) simulations are becoming increasingly common in educational settings, particularly in medical education. Advancing XR devices to enhance these simulations is a booming field of research. This study seeks to understand the value of a novel, non-wearable mixed reality (MR) display during interactions with a simulated holographic patient, specifically in taking a medical history. Twenty-one first-year medical students at the University of North Carolina at Chapel Hill participated in the virtual patient (VP) simulations. On a five-point Likert scale, students overwhelmingly agreed with the statement that the simulations helped ensure they were progressing along learning objectives related to taking a patient history. However, they found that, at present, the simulations can only partially correct mistakes or provide clear feedback. This finding demonstrates that the novel hardware solution can help students engage in the activity, but the underlying software may need adjustment to attain sufficient pedagogical validity.

## Introduction

Extended reality (XR) is an overarching term that encompasses augmented reality (AR), virtual reality (VR), mixed reality (MR), and everything in between [1]. The increased accessibility of immersive technologies has led to the increasingly common use of XR devices as a learning tool within medical education [2], raising questions about its efficacy and best uses. XR is a broader term encompassing technologies along a 'virtuality continuum' [3]. This spectrum offers immersive experiences ranging from interactions mostly existing in the natural environment to complete immersion in the virtual world [3]. AR is distinct from VR in that, while VR fully immerses one in a virtual environment, AR creates a mixed reality, allowing for interactions with both the digital and real world [4]. Differences between these forms of MR are reflected in their respective devices. Transparent head-mounted displays (HMDs), such as Microsoft HoloLens, are commonly used in AR to portray virtual content. In contrast, VR relies on fully immersive HMDs like the Oculus Rift [5]. However, HMDs are not required for visualization; recent advancements in 3D display, such as multi-view and light-field displays, have allowed holographic technology to play a more significant role in AR [6].

Initially utilized in military training and the gaming industry, MR devices are rapidly being adapted for the learning environment, and their use in medical education has grown significantly over the last decade [5]. MR devices are now used throughout the health sciences to assist in surgical procedures, learn human anatomy, and promote resource stewardship among trainees [7,8]. Multiple studies have found that medical educators and students support using MR-supplemental education over classical instruction [9,10].

A typical application of AR within medical education has been using virtual patients (VPs) [11]. The Association of American Medical Colleges (AAMC) defines VPs as 'a specific type of computer-based program that simulates real-life clinical scenarios; learners emulate the roles of healthcare providers to obtain a history, conduct a physical exam, and make diagnostic and therapeutic decisions' [12]. VPs rely on natural language processing (NLP) to converse with students. The integration of NLP software into VP encounters has evolved from keyword matching and context switching - where questions from learners are matched based on conversationally relevant keywords from the user [13] - to state-of-the-art NLP algorithms, thereby expanding the effectiveness of VP encounters as learning simulations [14]. VPs have also become more visually realistic with the improvement of rendering in game technology [15]. With the increasing effectiveness of VPs, multiple studies support the use of VPs in medical education as a means of learning and assessing anatomy, history-taking, physical exam skills, and clinical reasoning [14,16].

Compared to traditional standardized patients, VPs offer distinct advantages in that they are more uniform, cost-effective, and accessible [5,12]. Moreover, immersive technology engages learners, promoting meaningful engagement and student-centered learning [12,17]. Compared to traditional teaching modalities, immersive technologies have shown equivalent learning gains among students in the health sciences [18]. VP encounters have shown increased knowledge acquisition and retention among learners, and when compared to small-group teaching, these virtual encounters have led learners to feel more prepared to diagnose and treat patients [19,20]. Furthermore, the adaptability of simulations within medical education enhances student learning by scaffolding and promoting guided discovery [21]. With these potential benefits in mind, there are still unanswered questions and potential pitfalls of VP utilization, including: who maintains liability for poor feedback from the model, what biases may underlie their programming, and how this may detract from the benefit of true interpersonal interactions in medical education [12].

Most studies on immersive technologies and VP encounters in medical education utilize wearable or fully immersive technology [22,23]. Given the demonstrated benefits of VP encounters to student learning, this study investigated the use of a novel, non-wearable display in VP encounters that has yet to be evaluated in the literature.

## Materials and methods

The study asked volunteers (learners) to engage in a conversation with a digital avatar (virtual patient). The VP uses animation and blending to simulate the biomechanics of speech and other simple motions during the simulation. The VP is displayed on a freestanding 3D monitor (Looking Glass, Looking Glass Factory Inc., Brooklyn, NY). The Looking Glass (LG), a group-viewable 3D holographic display, is an emerging AR device uniquely combining light-field and volumetric display technology, offering an advantage over traditional holographic and 3D displays. While other devices require wearable technology or may have a limited number of view zones, meaning users can only view the holographic display from a few angles, the LG monitor enhances the user experience by rendering a group-viewable 3D display with multiple view zones [6,24]. This device was selected as it is sufficient to render the patient image and clinical environment but requires no wearable technology, unlike many other commercially available solutions for patient simulation [25]. A short video of the proprietary software and hardware can be viewed upon request to the corresponding author.

Interactive cases, designed to involve a call-and-response relationship between the learner and the VP, served as the basis for their interaction. The case scripts, involving disease state-specific dialog (appendicitis, chest pain, fatigue, lightheadedness, shortness of breath) and emotional state-specific dialog (depression, bipolar disorder, adjustment), were created by one author (Alexander) while a medical student and reviewed by several medical school students and faculty members with medical education and medical simulation backgrounds. The scripts were coded using a game narrative scripting language (Ink, Inkle Studios, Cambridge, UK) to test the game flow, then translated by a third-party vendor (BioMojo LLC, Cary, NC) and applied to the VP simulator in conjunction with an NLP algorithm to account for variability in user language choice. The cases and VP were then validated through alpha testing and feedback from second-year medical students.

Learners were recruited from the first-year class at a large public medical school in North Carolina. This demographic was selected as they are actively engaged in the curriculum at their institution, which teaches them patient interviewing skills such as those simulated in the interactive cases. Thus, they are neither experts nor content naïve. Learners engaged in a 25-minute session where they were briefly oriented to the simulator and allowed to engage in the cases freely. Learners followed on-screen prompts to ask the VP medical history questions; the VP returned a verbal response based on the underlying script and NLP algorithm. At the end of this session, learners completed an open-access brief questionnaire validated to measure engagement in interactive systems in higher education, adapted slightly to fit the context of this study [26], excluding questions relating to the learning environment, which did not apply to this simulation. This specific survey was selected due to a high degree of shared variance in an attempt to control for possible subjective interpretations of its Likert scale. Responses were collected in QualtricsXM (Qualtrics, Provost, UT), and descriptive statistics were then analyzed using STATA 17.0 (StataCorp LLC, College Station, TX).

## Results

Twenty-one students tested the simulation and completed the questionnaire. A summary of the Likert scale responses to the questionnaire can be found in Table 1. Students agreed most strongly with the statement that the interactive response system (IRS), the VP, helps them ensure they are "following the contents of the subject correctly during classes" (µ = 3.57, σ = 0.93). They agreed least with the statement that the "IRS allows them to correct mistakes or misunderstandings about the subject contents during classes" (µ = 2.81, σ = 1.08).

**Table 1:** Summary statistics of learner responses to the IRS questionnaire, adapted from Mingorance-Estrada ÁC et al., for this simulation. All participants (N=21) completed each question on the questionnaire. IRS: Interactive response system.

Statement	Totally Disagree (1)	Disagree (2)	Neutral (3)	Agree (4)	Totally Agree (5)	Mean (± SD)
Factor 2: Teaching-Learning Process						
The use of IRS [the virtual patient] improves my learning performance.	3	3	7	7	1	3.00 (± 1.14)
The IRS is used to find out the initial knowledge of the students.	1	1	10	8	1	3.33 (± 0.86)
Thanks to the IRS, I measure if I am following correctly the contents of the subject during classes.	1	2	3	14	1	3.57 (± 0.93)
IRS provides valuable information to improve your learning process.	2	2	5	9	3	3.43 (± 1.16)
The use of IRS is carried out by experienced professors to provide good feedback.	2	2	11	5	1	3.05 (± 1.21)
The use of the IRS is done at the end of the classes to review the contents explained during the session.	3	0	11	4	3	3.19 (± 1.17)
The continuous use of the IRS increases my class attendance.	2	4	10	3	1	2.85 (± 0.99)
Factor 3: Learning Assessment						
The use of IRS allows you to know and compare your colleagues’ answers with your own answers.	1	1	13	6	0	3.14 (± 0.73)
The use of IRS promotes regular study of the subject to be better prepared for classes.	1	2	8	8	3	3.38 (± 0.97)
The use of the IRS allows to correct mistakes or misunderstandings about the subject contents during the classes.	3	5	6	7	0	2.81 (± 1.08)
The use of IRS helps me to develop my comprehension on the contents I am working on.	3	1	3	12	2	3.43 (± 1.21)
The use of IRS improves motivation during classes.	1	2	9	8	1	3.29 (± 0.90)
The use of IRS improves the understanding of the contents explained in class.	3	1	4	11	2	3.38 (± 1.20)

## Discussion

This study sought to determine if MR devices, which offer an immersive but not enclosed experience and are increasingly common in medical education [2,5,9], can simulate patient interactions effectively as a valuable learning tool based on student experiences. These technologies have been favored in other studies and learning contexts for their scalability, availability, and internal consistency (reduction in human error) [12,17,18]. The results of the questionnaire indicate that this tool can help students assess their understanding of the concepts tested by the simulation (taking a patient history) and provide feedback on how to improve their learning performance. As a proof of concept, this demonstrates that non-wearable MR devices, such as the one tested here, can still be valuable tools in the armamentarium of medical educators. Participants indicated that they could engage with and derive value from their interactions with the VP in these simulations, even without using fully immersive technology.

The results also reveal some structural limitations of the technology as applied in this intervention, specifically the ability to correct mistakes, draw comparisons to one’s performance, and motivate students to engage with other learning technologies. These structural limitations suggest that the simulation, in its early version, was better suited for student assessment rather than learning. However, since these aspects of learning were not direct goals of its initial design, they could be readily addressed in future iterations of the simulation through gamification to promote deeper levels of engagement and provide a mechanism for comparison among users [27,28]. The rapidly evolving and adaptable nature of these technologies makes them potentially useful in various learning environments, which will continue to be the source of ongoing investigation.

There are multiple potential avenues for this technology to provide high-fidelity simulations in medical education and other fields, such as anatomy, nursing, dental, and medical subspecialty education [5,7,13,17,19,20]. These applications should be developed with the aforementioned limitations in mind and thoughtfully considered when determining if these technologies are best suited for a particular learning environment. Within medical education, improvements in the patient interface, including the development of a formal assessment structure, could provide a technological alternative to standardized patients, addressing longstanding concerns of bias and accuracy in traditional standardized patient encounters [29,30]. This technology also holds promise for more complex simulations, such as engaging with an interpreter or rendering discipline-specific, 3D images. These proposed alternatives and improvements should be the focus of development in anticipation of further research.

The results are limited by the utility of the validated questionnaire, which was designed specifically for activities integrated within the context of a course. However, this limitation would likely bias the findings toward the null, and further research conducted within a course context is expected to reveal more positive responses. It should also be noted that participation was not randomized, which introduces a potential selection bias, as participating students might find these technologies more interesting and useful than the average student. Future studies should either adjust the research setting to fit the validated tool or develop a validated mechanism specific to the proposed simulation and accompanying technology, with randomized participants. They should also consider head-to-head assessments of this technology against more conventional HMDs and other MR devices.

## Conclusions

In conclusion, these non-wearable 3D display technologies are promising solutions for educational simulations and have the potential to extend beyond the simulations evaluated in this study. This study found that medical student interactions with a VP through non-wearable MR devices can be valuable for students to practice patient interactions while applying medical knowledge and receiving feedback. As a proof of concept, this project demonstrates the potential of integrating MR into medical training as an accessible and student-centered learning tool. Further research and development will be necessary to fully address the current limitations of the technology and the study limitations described earlier.
